# Reference SVA insertion polymorphisms are associated with Parkinson’s Disease progression and differential gene expression

**DOI:** 10.1038/s41531-021-00189-4

**Published:** 2021-05-25

**Authors:** Abigail L. Pfaff, Vivien J. Bubb, John P. Quinn, Sulev Koks

**Affiliations:** 1 Perron Institute for Neurological and Translational Science, Perth, WA Australia; 2 Centre for Molecular Medicine and Innovative Therapeutics, Murdoch University, Perth, WA Australia; 3 Department of Pharmacology and Therapeutics, Institute of Systems, Molecular and Integrative Biology, University of Liverpool, Liverpool, UK

**Keywords:** Parkinson's disease, Genomics

## Abstract

The development of Parkinson’s disease (PD) involves a complex interaction of genetic and environmental factors. Genome-wide association studies using extensive single nucleotide polymorphism datasets have identified many loci involved in disease. However much of the heritability of Parkinson’s disease is still to be identified and the functional elements associated with the risk to be determined and understood. To investigate the component of PD that may involve complex genetic variants we characterised the hominid specific retrotransposon SINE-VNTR-Alus (SVAs) in the Parkinson’s Progression Markers Initiative cohort utilising whole genome sequencing. We identified 81 reference SVAs polymorphic for their presence/absence, seven of which were associated with the progression of the disease and with differential gene expression in whole blood RNA sequencing data. This study highlights the importance of addressing SVA variants and potentially other types of retrotransposons in PD genetics, furthermore, these SVA elements should be considered as regulatory domains that could play a role in disease progression.

## Introduction

Parkinson’s disease (PD) is the second most common neurodegenerative disease affecting 1% of the population over 60 years of age^1^. PD pathology is characterised by the loss of dopaminergic neurons from the substantia nigra and the formation of neuronal inclusions called Lewy bodies. The primary symptoms are related to motor dysfunction (bradykinesia, resting tremor, rigidity, and postural instability), however, individuals with PD can also develop a range of non-motor symptoms that include sleep disturbances, constipation and impaired olfaction^2^. The precise mechanisms underlying the development of PD are unknown. Genetic studies have played a key role in highlighting pathways involved in disease pathogenesis that include autophagy, mitophagy, and the innate immune system^3,4^. There are highly penetrant mutations in several genes that cause rare monogenic forms of PD in either an autosomal dominant (e.g., *SNCA* and *LRRK2*) or an autosomal recessive (e.g., *PRKN* and *PARK7*) manner^5^. However, monogenic forms of the disease contribute a small percentage to the total incidence of the disease, with the majority of cases classed as sporadic and likely caused by a complex interaction of genetic and environmental risk factors. Large-scale genome wide association studies (GWAS) have been crucial in identifying genetic risk factors of idiopathic disease, however, a large proportion of the disease heritability has yet to be identified^6,7^. In addition, many of the single nucleotide polymorphisms (SNPs) that are associated with disease development are in non-coding regions of the genome and their functional impact is often unknown. We aim to investigate the genetic component of PD further by studying other types of variation and their functional consequences and this study focuses on variation generated by a specific family of retrotransposons.

**Fig. 1:**
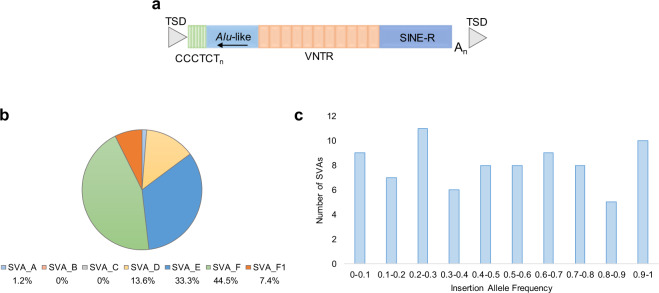
Structure, subtype classification and frequency of polymorphic reference SVAs in the PPMI cohort. **a** The structure of a canonical SVA consists of a (CCCTCT)_n_ hexamer repeat, an *Alu*-like region consisting of two antisense *Alu* fragments and an intervening unique sequence, a variable number tandem repeat (VNTR), a SINE-R domain and a poly A-tail. The length of an intact SVA can vary depending on the number of repeats present in the hexamer and VNTR domains. SVAs are flanked by target site duplications (TSDs). **b** The proportion of the 81 reference SVA RIPs that belong to each of the seven subtypes of SVA. **c** The number of reference SVA RIPs that belong in each category of insertion allele frequency.

SINE-VNTR-*Alus* (SVAs) are hominid specific retrotransposons and comprise ~2700 elements in the reference human genome^8^. SVAs are composite elements (domains outlined in Fig. 1a) consisting of seven subfamilies (A-F and F1) and are the evolutionarily youngest member of the non-long terminal repeat class of retrotransposons^9,10^. Members of the SVA family are still able to retrotranspose in the human genome^11^, and this ongoing mobilisation has led to insertions that are polymorphic in the human population for their presence or absence. Of those SVAs in the reference genome 77 have been reported in the dbRIP database as polymorphic in terms of their presence or absence^12,13^. There are also SVA insertions that are not in the reference genome and fourteen such polymorphic SVA insertions have been identified as the cause of diseases including X-linked dystonia parkinsonism (XDP), Neurofibromatosis type 1 and haemophilia B through mechanisms such as loss of function mutation, modulation of splicing and deletions at the site of insertion^14–16^. XDP is associated with a founder haplotype that contains a SVA in intron 32 of the *TAF1* gene, reduced expression of *TAF1* and alternative splicing and intron retention directed by the SVA insertion^17^. Polymorphic retrotransposons, including members of the SVA family, have been shown to regulate gene expression in a population specific manner and could have an impact on phenotypic differences^18^. In the literature retrotransposon insertion polymorphisms (RIPs) have also been identified to be in linkage disequilibrium (LD) with SNPs associated with a range of diseases through GWAS, further analysis of selected candidates identified six RIPs that could lead to disease through changes in gene regulation^19^.

Our study focused on the potential role of reference SVA RIPs established in the population in the risk and progression of PD. To address this we utilised whole genome sequence, transcriptomic and clinical data from the Parkinson’s Progression Markers Initiative (PPMI) which was established to identify PD progression markers to help understand disease aetiology and ultimately aid in the development of novel therapeutics^20^. The PPMI is a longitudinal study collecting clinical and imaging data and biospecimens from PD subjects, healthy controls and those subjects classified as SWEDD (scans without evidence of dopaminergic deficit)^21^. Using the extensive dataset from the PPMI cohort, we identified 81 SVAs that were polymorphic for their presence or absence in the reference genome. Seven of the identified SVA RIPs were associated with PD progression and subsequent analysis of these seven RIPs correlated specific genotypes with changes in gene expression in the blood.

## Results

### Characterisation of reference SVA RIPs in the PPMI cohort

The PPMI cohort, whose whole genome sequence data were analysed in this study, consisted of 179 healthy controls, 371 PD and 58 subjects classified as SWEDD. The subjects from each group showed similar demographics in terms of age and sex (Table 1) and all subjects included reported race as white. In the 608 subjects analysed using the structural variant caller Delly (https://github.com/dellytools/delly), 83 SVAs in the human reference genome were identified as absent in at least one genome compared to the reference (hg38) (Supplementary Table 1). Two of these were detected as absent in all genomes analysed and therefore were not classified as polymorphic in this cohort. The same individuals were also analysed using mobile element locator tool deletion (MELT-del)^22^ which can detect polymorphic reference retrotransposons. MELT-del only detected 50 reference SVAs as absent compared to the 83 by Delly. DNA samples from the PPMI cohort were obtained for validation purposes of the variants detected. Three SVAs were amplified using PCR, two of which (SVA_27 and SVA_32) were detected by both Delly and MELT-del and the third (SVA_24) was only detected by Delly (see Supplementary Fig. 1 for representative gel images). The genotypes of the three SVAs called by Delly were in 98.4–99.4% agreement with the PCR genotypes compared to 91.5–95.6% for those called by MELT-del (Supplementary Table 2). Genotypes called by the tool Delly demonstrated greater accuracy and a higher number of SVA RIPs were detected compared to MELT-del, therefore Delly was chosen as the most suitable tool for this analysis.

**Table 1:** Demographics of the PPMI subjects.

Variable	PPMI subjects included in genome wide genotyping of reference SVAs		
	PD (n = 371)	Healthy controls (n = 179)	SWEDD (n = 58)
Sex			
Male	241 (65%)	117 (65%)	38 (66%)
Female	130 (35%)	62 (35%)	20 (34%)
Age			
Mean (min, max)	62.0 (33.5–84.9)	61.3 (30.6–82.7)	60.7 (38.3–78.8)
Family history			
1^st^ degree family member with PD	51 (14%)	0 (0%)	13 (24%)
Other family member with PD	43 (12%)	10 (6%)	5 (9%)
No family member with PD	276 (74%)	169 (94%)	39 (67%)

Of the 81 SVAs RIPs detected by Delly, 28 (34.6%) had not been reported previously in the dbRIP track available on UCSC genome browser. The majority, 85.2%, of the SVA RIPs belonged to subtypes E, F and F1 (Fig. 1b), which was consistent with these human specific subfamilies being the most recently active^9^. The SVA RIPs showed a wide range of insertion allele frequencies from 0.0008-0.994 (Fig. 1c). Using logistic regression and Bonferroni correction for multiple testing there were no SVA RIPs significantly associated with PD when comparing control and PD subjects in this cohort (Supplementary Table 1). Tagging SNPs (r^2^ > 0.8) were identified for 76 (93.8%) of the SVA RIPs and were used to act as a proxy for the SVA genotype, enabling the evaluation of these elements in the largest PD and healthy control dataset available from the recent GWA meta-analysis performed by Nalls et al^6^. In the summary statistics from this meta-analysis, 75 of the SVA RIP tagging SNPs were present (Supplementary Table 3). One of these SNPs (rs55653937) was reported as associated with PD at genome wide significance (p = 1.64×10^−19^, β = −0.2488) and was in strong LD (r^2^ = 0.994) with SVA_67 located 12 kb upstream of the *KANSL1* gene. SVA_67 was not significantly associated with PD in the PPMI cohort, however the direction of the effect was the same as rs55653937 (OR 0.91, 95% CI 0.66-1.24).

### Reference SVA RIPs are associated with progression markers of PD in the PPMI cohort

**Fig. 2:**
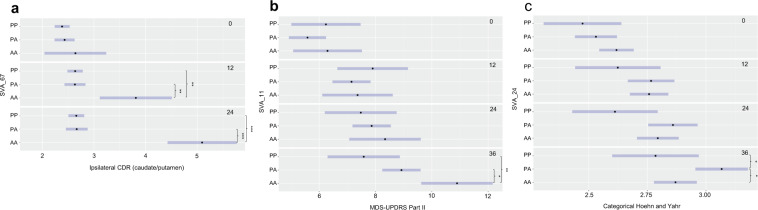
Polymorphic SVAs were associated with markers of PD progression in the PPMI cohort. **a** PD individuals with the AA genotype of SVA_67 had a significantly higher ipsilateral caudate/putamen CDR at both 12 and 24 months compared to those with PA and PP genotypes. **b** PD individuals with the AA genotype of SVA_11 had a significantly higher MDS-UPDRS part II score at 36 months compared to those with PA and PP genotypes. **c** PD individuals with the PA genotype of SVA_24 had a significantly higher Hoehn and Yahr score at 36 months compared to those with AA and PP genotypes. 95% confidence intervals are represented by the blue bar. *p < 0.05, **p < 0.01 and ***p < 0.001 (Tukey adjusted p values are reported).

The PPMI cohort has extensive clinical and phenotypic data available at four time points, baseline (0 months) and subsequent measurements at 12, 24 and 36 months. This data includes motor, cognitive and olfactory testing, dopamine transporter (DAT) imaging assessments and analysis of markers in biospecimens. Analysis of the 81 SVA RIPs identified seven associated with features of PD (Table 2) at 12 months or later. In Table 2 the FDR corrected p values are reported for the linear mixed effects model and Tukey adjusted p values for the pairwise analysis addressing the genotypic effect. The nearest gene to each of these SVAs are reported in Table 3 and for chromosomal loci see Supplementary Table 1. Four of the SVA RIPs (36, 47, 67 and 79) in Table 3 were associated with changes in the count density ratios (CDR) of the caudate/putamen, which had been calculated from DaTscan single photon emission computed tomography (SPECT) imaging. Figure 2a highlights this for SVA_67, located upstream of the *KANSL1* gene, showing PD subjects with AA genotype of SVA_67 had a significantly higher mean caudate/putamen CDR (3.81, 95% CI 3.12–4.51) than those with both PA (2.63, 95% CI 2.43–2.83, *p* = 0.004) and PP (2.64, 95% CI 2.49–2.79, *p* = 0.004) genotypes at 12 months. The AA PD subjects also had a significantly higher mean (5.10, 95% CI 4.43–5.76) at 24 months compared to PA (2.67, 95% CI 2.46–2.88, *p* < 0.001) and PP (2.66, 95% CI 2.51–2.81, *p* < 0.001). The remaining three SVA RIPs (7, 11, and 24) in Table 3 were associated with the following measurements reflecting the progression of PD: Hoehn and Yahr staging and the Movement Disorder Society – Unified Parkinson’s Disease Rating Scale (MDS-UPDRS). For example, the presence of SVA_11 was significantly associated with a lower mean MDS-UPDRS part II score at 36 months (Fig. 2b) when comparing PP (7.6, 95% CI 6.3–8.9) and PA (8.9, 95% CI 8.2–9.6) genotypes to AA (10.9, 95% CI 9.6–12.2, *p* = 0.001 and *p* = 0.02). SVA_24 was significantly associated with changes in the categorical Hoehn and Yahr stage in PD subjects (Fig. 2c). Those with a PA genotype had a higher mean Hoehn and Yahr stage at 36 months (3.07, 95% CI 2.96–3.18) compared to AA (2.87, 95% CI 2.78–2.96, *p* = 0.02) and PP genotypes (2.78, 95% CI 2.60–2.97, *p* = 0.03).

**Table 2:** SVAs RIPs that are associated with clinical features and markers of progression of PD in the PPMI cohort. The FDR corrected p value from the linear mixed-effects model is reported for each SVA. The mean and 95% confidence intervals (CI) are reported for each genotype at each visit with data available and Tukey adjusted p values from the pairwise analysis are shown for each pair of genotypes. (Bold highlights significant p values in pairwise analysis).

SVA ID	Associated feature	Time of visit (months)	FDR corrected p value from lme model	AA mean (95% CI)	PA mean (95% CI)	PP mean (95% CI)	AA vs PA *p*-value	AA vs PP *p*-value	PA vs PP *p*-value
SVA_7	MDS-UPDRS Part III	0	0.04	24.5 (9.5–39.5)	20.9 (18.5–23.3)	21.0 (19.8–22.3)	0.88	0.89	0.99
	Score (OFF)	12		28.5 (13.5–43.5)	23.9 (21.4–26.4)	25.6 (24.2–26.9)	0.82	0.92	0.49
		24		27.0 (12.1–42.0)	26.0 (23.4–28.7)	27.5 (26.1–29.0)	0.99	0.99	0.57
		36		10.0 (−4.96–25.0)	28.4 (25.7–31.1)	29.5 (28.1–31.0)	**0.05**	**0.03**	0.75
SVA_11	MDS-UPDRS Part II	0	0.003	6.3 (5.1–7.52)	5.6 (4.9-6.3)	6.2 (5.0–7.5)	0.57	>0.99	0.62
	Score	12		7.4 (6.1–8.6)	7.2 (6.5–7.8)	7.9 (6.7–9.2)	0.95	0.82	0.55
		24		8.3 (7.1–9.6)	7.9 (7.2–8.5)	7.5 (6.2–8.8)	0.79	0.61	0.85
		36		10.9 (9.6–12.2)	8.9 (8.2–9.6)	7.6 (6.3–8.9)	**0.02**	**0.001**	0.16
SVA_24	Categorical Hoehn	0	0.04	2.62 (2.55–2.69)	2.53 (2.44–2.62)	2.47 (2.31–2.64)	0.30	0.26	0.82
	& Yahr	12		2.76 (2.68–2.84)	2.77 (2.67–2.87)	2.62 (2.44–2.81)	0.99	0.38	0.37
		24		2.79 (2.71–2.88)	2.86 (2.75–2.96)	2.61 (2.43–2.79)	0.62	0.18	0.06
		36		2.87 (2.78–2.96)	3.07 (2.96–3.18)	2.78 (2.60–2.97)	**0.02**	0.70	**0.03**
SVA_36	Ipsilateral count	0	0.02	2.39 (1.98–2.79)	2.40 (2.22–2.58)	2.43 (2.27–2.59)	>0.99	0.98	0.96
	density ratio	12		3.36 (2.93–3.79)	2.55 (2.36–2.74)	2.64 (2.48–2.81)	**0.003**	**0.007**	0.77
	(caudate/putamen)	24		3.72 (3.28–4.17)	2.63 (2.44–2.82)	2.68 (2.51–2.86)	**<0.0001**	**0.0001**	0.91
SVA_47	Ipsilateral count	0	0.008	2.40 (2.29–2.52)	2.69 (2.15–3.23)	2.53 (1.27–3.78)	0.57	0.98	0.97
	density ratio	12		2.64 (2.51–2.76)	3.39 (2.85–3.93)	2.89 (1.40–4.38)	**0.02**	0.94	0.81
	(caudate/putamen)	24		2.66 (2.54 –2.79)	4.44 (3.87–5.01)	3.18 (1.18–5.19)	**<0.0001**	0.87	0.46
SVA_67	Ipsilateral count	0	1.97 × 10^-6^	2.64 (2.04–3.24)	2.43 (2.24–2.62)	2.38 (2.24–2.53)	0.79	0.69	0.91
	density ratio	12		3.81 (3.12–4.51)	2.63 (2.43–2.83)	2.64 (2.49–2.79)	**0.004**	**0.004**	>0.99
	(caudate/putamen)	24		5.10 (4.43–5.76)	2.67 (2.46–2.88)	2.66 (2.51–2.81)	**<0.0001**	**<0.0001**	>0.99
SVA_79	Contralateral count	0	0.03	–	3.26 (2.75–3.78)	2.87 (2.76–2.98)	–	–	0.14
	density ratio	12		–	2.40 (1.87–2.93)	2.93 (2.82–3.04)	–	–	0.06
	(caudate/putamen)	24		–	2.18 (1.67–2.69)	2.93 (2.81–3.04)	–	–	**0.005**

**Table 3:** Differentially expressed genes significantly associated with the genotype of SVA RIPs at baseline (0 months) and 36 months follow up visit. The number of genes significantly differentially expressed (FDR corrected p < 0.1) are reported for each SVA RIP genotype followed by the most significant gene in brackets.

SVA ID	Nearest gene to SVA	No. of significantly differentially expressed genes at baseline			No. of significantly differentially expressed genes at 36 month visit			Tagging SNP for SVA reported as an eQTL in GTEx portal	
		PP v AA	PP v PA	PA v AA	PP v AA	PP v PA	PA v AA	Whole blood	Other tissues
SVA_7	*OR14I1* (4 kb downstream)	0	686 (*EVPL*)	2 (*HBA2*)	1 (AC003102.1)	28 (*RAB3B*)	3 (AC003102.1)	N	Y
SVA_11	*CASP8* (intron)	1 (*FLACC1*)	0	64 (*IGSF3*)	36 (*IGLV7-46*)	15 (*IGLV7-43*)	5 (*IFI27*)	no tag r^2^ > 0.8	no tag r^2^ > 0.8
SVA_24	*HLA-A* (8 kb upstream)	4 (*HLA-A*)	13 (*IGKV1-16*)	3 (*HLA-A*)	10 (*HLA-A*)	7 (*SERINC4*)	13 (*HLA-A*)	SNP not present	SNP not present
SVA_36	*NUPR2* (30 kb upstream)	1467 (*TSPAN11*)	5 (*PCSK1N)*	207 (*FAM155B*)	1474 (*RPL31*)	36 (*ADGRG7*)	823 (*GIMAP2*)	Y	Y
SVA_47	*RAD23B* (10 kb upstream)	0	0	12 (*IL32*)	75 (AC01177.3)	42 (*SNORC*)	11 (*PLAU*)	N	N
SVA_67	*KANSL1* (12 kb upstream)	4 (*PLEKHM1*)	7 (*ARL17A*)	0	22 (AC120194.1)	25 (*PLEKHM1*)	13 (AC120194.1)	Y	Y
SVA_79	*PFDN4* (30 kb downstream)	na	1 (*RAB3B*)	na	na	0	na	N	N

### The genotype of specific SVA RIPs is associated with differential gene expression

Transcriptomic data from whole blood was utilised to determine if the seven SVA RIPs associated with PD progression (Table 3) had any influence on gene expression. RNA sequencing data from whole blood was available at baseline (0 months) and four additional time points (6, 12, 24 and 36 months). In the analysis, all subjects in the cohort were used to compare gene expression profiles between subjects with the three different genotypes (PP, PA and AA) based on the presence or absence of the specific SVA RIP with statistical analysis performed at 0 and 36 months. For all of the seven SVA RIPs analysed, at least one genotype at one time point was associated with differential gene expression (Table 3). The number of genes whose differential expression was significantly associated with specific SVA genotypes (FDR < 0.1) at 0 and 36 months is summarised in Table 3 and the gene with the lowest adjusted *p* value is shown in brackets. Genotypes of SVA_7 and SVA_36 were associated with changes in gene expression of hundreds or thousands of genes at many different loci. Whereas the effects of other SVA RIPs (24 and 67) were restricted to a smaller number of genes (Table 3), many of which were located at the SVA locus, and several of those genes were affected at both time points analysed. However, one SVA RIP demonstrated a narrower effect on gene expression with only the expression of one gene (*RAB3B*) associated with SVA_79 genotype. The effect of the SVA RIPs on gene expression varied between the different elements and below we highlight specific examples of genes or clusters whose expression was associated with multiple SVA genotypes and/or at both time points at which statistical analysis was performed.

**Fig. 3:**
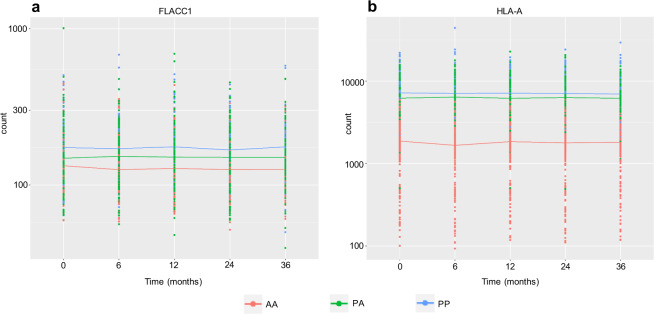
The genotype of reference SVAs was associated with differential gene expression. **a** At 0 months a 1.3 fold change in expression of *FLACC1* was significantly associated with SVA_11 genotype when comparing PP to AA (FDR *p* = 0.04) and at 36 months a 1.4 fold change in expression when comparing PP to AA (FDR *p* = 2.22 × 10^−5^). **b** At 0 months a 3 fold change in expression of *HLA-A* was significantly associated with SVA_24 genotype when comparing PA to AA (FDR *p* = 1.90 × 10^−78^) and a 3.7 fold change in expression when comparing PP to AA (FDR *p* = 6.22 × 10^−51^). At 36 months a 2.5 fold change in expression of *HLA-A* was significantly associated with SVA_24 genotype when comparing PA to AA (FDR *p* = 7.51 × 10^−60^) and a 3.6 fold change in expression when comparing PP to AA (FDR *p* = 2.49 × 10^−38^). *n* = 608.

The expression of *FLACC1* was significantly different for individuals with PP genotype compared to AA for SVA_11 (located in an intron of *CASP8*) at 0 (FDR *p* = 0.04) and 36 months (FDR *p* = 2.22 × 10^−5^) (Fig. 3a). *FLACC1* is adjacent to *CASP8* in the genome and SVA_11 is 3.5 kb downstream of its 3’UTR. The expression of *HLA-A* was repeatedly the most significant gene to be associated with SVA_24 (located 8 kb upstream of *HLA-A*) genotypes at 0 and 36 months (Table 3). At both 0 and 36 months the subjects with PP genotypes had significantly higher *HLA-A* expression than those with AA genotypes (FDR *p* = 6.22 × 10^−51^ and *p* = 2.49 × 10^−38^) (Fig. 3b). Those with PA genotypes also showed significantly higher *HLA-A* expression than AA genotypes at 0 and 36 months (FDR *p* = 1.90 × 10^−78^ and *p* = 7.51 × 10^−60^), however, there was no significant difference when comparing PP and PA genotypes (Fig. 3b).

**Fig. 4:**
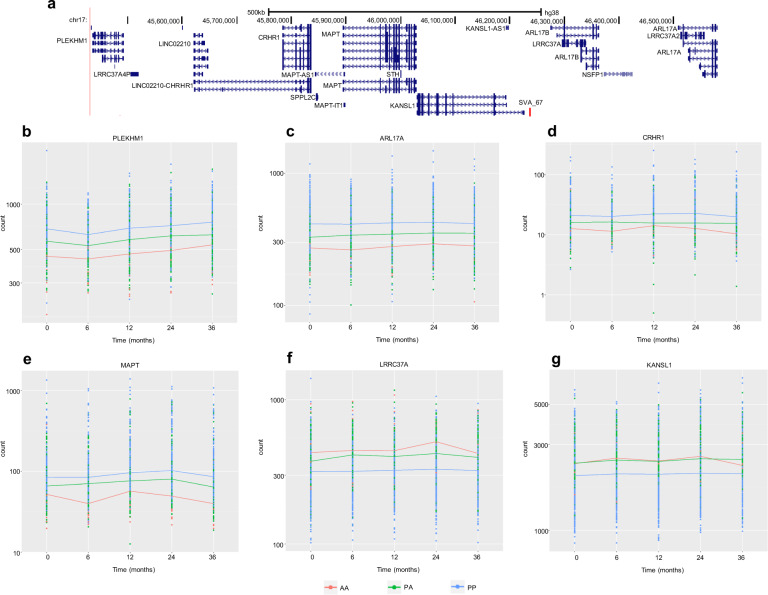
The expression of several genes at the 17q21.31 locus were associated with SVA_67. **a** A schematic modified from UCSC genome browser of the 1.15 Mb region on chromosome 17 that contains six of the genes whose expression was associated with SVA_67. **b** At 0 months a 1.5 fold change in expression of *PLEKHM1* was significantly associated with SVA_67 genotype when comparing PP to AA (FDR *p* = 2.38 × 10^−6^) and a 0.8 fold change in expression when comparing PA to PP (FDR *p* = 2.40 × 10^−9^). At 36 months a 1.4 fold change in expression of *PLEKHM1* was significantly associated with SVA_67 genotype when comparing PP to AA (FDR *p* = 0.008) and a 0.8 fold change in expression when comparing PA to PP (FDR *p* = 1.01 × 10^−6^). **c** At 0 months a 1.6 fold change in expression of *ARL17A* was significantly associated with SVA_67 genotype when comparing PP to AA (FDR *p* = 8.72 × 10^−5^) and a 0.8 fold change in expression when comparing PA to PP (FDR *p* = 1.21 × 10^−9^). At 36 months a 1.5 fold change in expression of *ARL17A* was significantly associated with SVA_67 genotype when comparing PP to AA (FDR *p* = 0.006) and a 0.8 fold change in expression when comparing PA to PP (FDR *p* = 0.003). **d** At 0 months a 1.8 fold change in expression of *CRHR1* was significantly associated with SVA_67 genotype when comparing PP to AA (FDR *p* = 7×10^−^^4^) and a 0.7 fold change in expression when comparing PA to PP (FDR *p* = 1.47 × 10^−8^). At 36 months a 2.1 fold change in expression of *CRHR1* was significantly associated with SVA_67 genotype when comparing PP to AA (FDR *p* = 2.5 × 10^−4^) and a 0.7 fold change in expression when comparing PA to PP (FDR *p* = 1.87 × 10^−5^). **e** At 0 months a 0.8 fold change in expression of *MAPT* was significantly associated with SVA_67 genotype when comparing PA to PP (FDR *p* = 0.001). At 36 months a 2.3 fold change in expression of *MAPT* was significantly associated with SVA_67 genotype when comparing PP to AA (FDR *p* = 0.002) and a 0.7 fold change in expression when comparing PA to PP (FDR *p* = 0.01). **f** At 0 months a 1.2 fold change in expression of *LRRC37A* was significantly associated with SVA_67 genotype when comparing PA to PP (FDR *p* = 0.03). At 36 months 1.2 fold change in expression of *LRRC37A* was significantly associated with SVA_67 genotype when comparing PA to PP (FDR *p* = 0.002). **g** At 36 months 1.2 fold change in expression of *KANSL1* was significantly associated with SVA_67 genotype when comparing PA to PP (FDR *p* = 0.06). *n* = 608.

SVA_67 at the chromosomal locus 17q21.31 was associated with differential expression of multiple genes, including six in a 1.15 Mb region centred around the SVA RIP (Fig. 4a). At baseline when comparing PP and AA genotypes three (*PLEKHM1* (FDR *p* = 2.38 × 10^−6^), *ARL17A* (FDR *p* = 8.72 × 10^−5^) and *CRHR1* (FDR *p* = 7 × 10^−4^)) out of the four significantly associated genes were located in this region as were five (*PLEKHM1* (FDR *p* = 2.40 × 10^−9^), *ARL17A* (FDR *p* = 1.21 × 10^−9^)*, CRHR1* (FDR *p* = 1.47 × 10^−8^)*, MAPT* (FDR *p* = 0.001) and *LRRC37A* (FDR *p* = 0.03)) out of seven genes whose expression was significantly different when comparing PP to PA genotypes (Fig. 4b–f). Extending the analysis to expression data at 36 months there were 22 genes whose expression was significantly different when comparing PP and AA genotypes of SVA_67, 4 of which were located in the 1.15 Mb region (*PLEKHM1* (FDR *p* = 0.008), *ARL17A* (FDR *p* = 0.006), *CRHR1* (FDR *p* = 2.5 × 10^−4^) *and MAPT* (FDR *p* = 0.002)) (Fig. 4b–e). These same four genes as well as two others (*LRCC37A* (FDR *p* = 0.002) and *KANSL1* (FDR *p* = 0.06)) in this genomic region were also differentially expressed when comparing PP and PA genotypes at 36 months (Fig. 4b–g). Four of the genes in this region whose expression was associated with SVA_67 (*PLEKHM1, ARL17A, CRHR1* and *MAPT*) all showed higher expression in individuals with PP genotypes and the individuals with the lowest expression had an AA genotype. This was in contrast with the levels of expression of *LRCC37A* and *KANSL1* where the opposite pattern was observed.

For the seven SVAs analysed for their association with differential gene expression six had tagging SNPs available. The Genotype-Tissue Expression (GTEx) portal (https://gtexportal.org/home/)^23^ and the six SVA tagging SNPs were used to extend the findings above. Five of the tagging SNPs were located in the GTEx portal, three of which were identified as expression quantitative trail loci (eQTLs) in several tissues (Table 3). The tagging SNP for SVA_67 was identified as an eQTL in 49/54 tissues, including 13 brain regions, and was associated with expression of over 30 genes, which included the six genes in Fig. 4.

## Discussion

This study incorporated genetic, clinical and gene expression data from the PPMI cohort to evaluate the role of reference SVAs in PD, identifying elements associated with disease progression that can modify the transcriptome. In this analysis, we characterised novel variation in the human genome identifying 81 reference SVA RIPs. In the PPMI cohort we did not identify any SVAs that were associated with disease risk. However, in the literature RIPs have been reported as candidate causative variants at known disease associated loci by identifying those in strong LD with trait associated SNPs^19,24^. Utilising this approach we identified one SVA RIP in our cohort in strong LD with a SNP associated with PD risk at genome wide significance. This SVA (SVA_67) is located in a 1.8 Mb region of high LD on chromosome 17 where there are two predominant haplotypes (H1 and H2) and the presence of SVA_67 is part of H1 with the SVA absent in H2. H1 is associated with increased risk of developing PD in multiple studies^25–27^. This region encompasses several genes and includes *MAPT* in which mutations can cause the neurodegenerative diseases frontotemporal dementia with parkinsonism and progressive supranuclear palsy^28,29^. However, it remains unclear which variant or gene within the H1 haplotype is responsible for the increased risk to PD and our study has highlighted additional types of variation to be considered at this locus. Functional work would be required to further explore the influence of SVA _67 at this locus and to identify the functional risk allele in this complex region.

The role of SVAs in the progression of PD has not been thoroughly addressed in the literature and here we identified seven SVAs associated with the progression of PD (Table 2). Clinical scales such as the Hoehn and Yahr and the MDS-UPDRS are used to measure disease severity and three of the SVA RIPs were associated with significant differences in these scores between the different genotypes of the insertions. The remaining four SVA RIPs were associated with changes observed in DaTscan SPECT analysis, specifically the ratio of caudate to putamen CDR (Table 2). DaTscan SPECT utilises a radioligand that binds to the DAT that is present on the presynaptic membrane of nigrostriatal neurons. The loss of the DAT from the striatum is characteristic of PD, correlated with the loss of dopaminergic neurons from the substantia nigra and used to distinguish PD from other neurodegenerative parkinsonisms such as essential tremor^30^. The reduction of striatal DAT correlates with symptom severity and changes over the course of the disease, therefore this can be used to monitor dopaminergic degeneration^31,32^. The caudate/putamen ratio is a measure of the anterior to posterior gradient of dopaminergic loss and increases in this gradient were associated with specific SVA RIP genotypes. The SVAs associated with these changes may not be directly causing these alterations to the gradient of dopaminergic loss but may be involved in modifications to the disease course that are measured by these features of DaTscan SPECT.

We have previously shown that SVAs can affect gene expression in in vitro and in vivo models using reporter gene assays^8,33^. More recently, studies have integrated genetic data regarding SVA RIPs and RNA sequencing to identify those elements that regulate transcription^18,19^. The SVA RIPs associated with the progression of PD had a significant impact on the transcriptome and this could be one of the mechanisms by which the SVAs are exerting an effect on PD progression. Genes at both the SVA RIP locus and distant regions of the genome, including different chromosomes, were associated with specific SVA RIP genotypes. For example, SVA_24 was most significantly associated with robust changes of expression of its nearest gene (*HLA-A*) and SVA_67 was associated with differential expression of a cluster of neighbouring genes. In contrast, SVA_36 was associated with changes in expression of thousands of genes at many different loci including those on different chromosomes to the SVA RIP. These differences observed could be due to the SVA insertion itself, the genomic architecture at each particular locus or a combination of both. SVAs could influence gene expression through multiple mechanism such as the recruitment of transcription factors, the interaction of SVAs with distant promoters through 3D chromatin structure and intronic insertions affecting mRNA splicing. Regulatory domains can function in a tissue-specific manner and therefore we utilised the GTEx database^23^ to evaluate the potential for the SVAs to act in other tissues using their proxy SNPs. Tagging SNPs for five out of the seven SVAs were located in the GTEx database and three of these were identified as eQTLs in multiple tissues (Table 3), including different regions of the brain such as the caudate, putamen, substantia nigra and cerebellum. This suggests the SVAs (7, 36 and 67) could also be influencing gene expression in other tissues in addition to whole blood demonstrated in this study.

The strength of this study was the integration of different types of data to evaluate the role of reference SVAs, which have yet to be addressed in detail, in PD risk and progression. The richness of the PPMI longitudinal data available is a clear benefit to this study and is a very valuable resource. The high quality WGS data from PPMI enabled accurate genotyping of the SVAs and DNA samples available for the same individuals was important for validation. However, the study was limited to a single family of retrotransposons and did not address those belonging to the *Alu* and L1 families, which would be important to evaluate in future work. Another limitation is the small number of samples for identifying SVAs involved in the risk of developing PD, however, the tagging SNP approach enabled the evaluation of these elements in much larger well-powered case-control cohorts. Using this systematic approach to focus our analysis on SVAs in PD we identified novel genetic variation in the PPMI cohort and specific SVA RIPs that were associated with dopaminergic degeneration. We were able to demonstrate these elements have a regulatory function and could be influencing the disease course through modulation of gene expression.

## Methods

### Identifying SVAs polymorphic for their presence/absence in the reference genome from whole genome sequencing data from the PPMI cohort

Whole genome sequencing data in bam file format aligned to hg38 were obtained from the PPMI. The use of data from PPMI was approved by the Human ethics committee at Murdoch University (2020/040). Written and informed consent was obtained by PPMI when participants were enrolled. The structural variant caller Delly2 (https://github.com/dellytools/delly)^34^, with default settings, was used to genotype structural variants in 608 individuals (179 healthy controls, 371 PD and 58 SWEDD) from the PPMI cohort. Those structural variants located within the coordinates of the human specific SVAs, available on the UCSC genome browser (https://genome.ucsc.edu/)^35^, ±100bp were extracted. Structural variants classified as deletions in these regions were inspected manually to determine if the break points were consistent with the reported coordinates of the reference human-specific SVAs. Traces from IGV over the SVA_32 region are shown in Supplementary Fig. 2 to demonstrate three different genotypes of this SVA. As a comparison MELT-del was also used to identify and genotype polymorphic reference SVAs in the WGS data and as Delly out performed MELT-del (see results section and Supplementary Table 2) genotypes called by Delly were taken forward in the analyses. Association analysis of the 81 SVA RIPs (identified by Delly) with PD was performed using logistic regression adjusted for age, gender and family history of PD in PLINK (v1.07)^36^, using data from the control and PD samples only, and *p* values adjusted for multiple testing (Bonferroni correction).

VCF files for the PPMI samples, generated by Project 188, were downloaded from the PPMI website (www.ppmi-info.org/data). SNPs with a minor allele frequency greater than 0.001 located in 250 kb on either side of the 81 SVA RIPs were extracted and PLINK (v1.07) was used to identify those SNPs in strong LD with the RIPs (*r*^2^ > 0.8). For each SVA RIP the SNP in strongest LD was selected and searched for in the summary statistics from Nalls et al.^6^ to determine if this proxy SNP for the SVA RIP genotype was associated with PD risk.

### PCR validation of selected reference SVA in DNA samples from the PPMI cohort

SVA_24 and SVA_32 were amplified using KOD Hot start polymerase (Novagen) under standard conditions with the addition of 1 M betaine and primers flanking the SVA insertion to amplify the absent (empty site) and present (filled site) alleles (SVA_24: For 5′ TCCCAGGGTCAGAAAAGATG 3′ Rev 5′ TAAGCCCCACTGTGATAGGG 3′, SVA_32: For 5′ ACCTGCTCTCCTATGATCATCT 3′, Rev 5′ AATCGAAGCAAATGGAGGCC 3′). For SVA_27 three primers were designed for the amplification of the empty site and the 3′ junction of the insertion in separate reactions using GoTaq G2 Hot Start polymerase (Promega) under standard conditions (SVA_27: For 5′ AGATTTCAGCAATGTCCTCCA 3′, Rev 5′ CAACAAACCAGGCACACACT 3′, internal SVA primer 5′ TGTTTATCTGCTGACCTTCCC 3′). The resulting PCR products were analysed by agarose gel electrophoresis or using the MultiNA (Microchip Electrophoresis System for DNA/RNA Analysis) to genotype each samples for the presence or absence of insertion based on the product size.

### Association analysis of SVA RIPs with clinical features and progression markers of PD in the PPMI cohort

For longitudinal analysis, a linear mixed-effects model combined with FDR correction measured the changes in phenotype scores during follow-up visits related to the 81 SVA RIPs in the PPMI cohort. The linear mixed-effects model used was: lme(response~R_SVA_XXX * months, random = ~1|PATNO, na.action=na.omit, data=PD) using the nlme package in R. Categorical variables were converted into numeric variables by using “as.numeric” option in R. Longitudinal data were obtained from PPMI (www.ppmi-info.org/data) and the most recently compiled files (PPMI_Baseline_Data_02Jul2018 and PPMI_Year_1-3_Data_02Jul2018) at the time of download were used. Altogether, 111 different clinical variables (Supplementary Table 4) were analysed in the PD subjects from four visits at baseline (0), 12, 24 and 36 months for the majority of the data points (data relating to the DaTscan SPECT imaging was not available at 36 months). The *p* values from the linear mixed-effects model were corrected for the number of features analysed and the FDR corrected *p-value* is reported in Table 2. A pairwise analysis was then performed using estimated marginal means on the SVAs that were significant after FDR correction for an effect size comparison with Tukey adjusted *p-value* for family-wise error rate reported in Table 2. The number of PD subjects analysed at each time point for each genotype of the seven significant SVAs in the linear mixed-effects model and pairwise comparison are reported in Supplementary Table 5.

### Analysis of SVA RIP genotype and gene expression in blood RNA-sequencing data from the PPMI cohort

In order to evaluate the effect of SVA RIP genotypes on the gene expression profile, differential whole transcriptome analysis based on whole blood RNA sequencing data (obtained from www.ppmi-info.org/data) was performed on all subjects (healthy controls, PD and SWEDD). The DESeq2 package in R was used to detect statistically significant differences in the gene expression profiles between the different genotypes of the seven SVAs associated with clinical features of PD at baseline (0 months) and 36 months and p values were FDR adjusted. The analysis was repeated in the PD subjects alone and a similar pattern of gene expression was observed (Supplementary Fig. 3). The SNPs were queried using the GTEx web browser portal (available at https://gtexportal.org/home/)^23^ to complement and extend this analysis by utilising the tagging SNPs identified for 6 out of the 7 SVAs from Table 3 to determine if they are eQTLs in a range of tissues. The GTEx project is an ongoing effort to build a comprehensive public resource to study tissue-specific gene expression and regulation. The GTEx Portal provides open access to data including gene expression, QTLs, and histology images.

### Reporting summary

Further information on research design is available in the Nature Research Reporting Summary linked to this article.

## Supplementary information

Supplementary Information

Reporting Summary

## Data Availability

The data used in this study was obtained from the Parkinson’s Progression Markers Initiative (PPMI) database (www.ppmi-info.org/data). Access to sequencing and phenotype data is controlled by PPMI. The datasets used in this study were whole genome sequencing bam files (Hg38), VCF files (Project 188), RNA sequencing from whole blood from five time points (counts data from Phase 1 and 2 RNA-seq aligned to hg19) and longitudinal clinical and phenotypic data (PPMI_Baseline_Data_02Jul2018 and PPMI_Year_1-3_Data_02Jul2018). The data used for the analyses described in this manuscript were obtained from the GTEx Portal (https://gtexportal.org/home/). The GTEx Analysis V8 was used, which provides access to de-identified gene expression data, QTL and histology images. The GTEx Analysis V8 dataset used in this project is publicly available on the GTEx portal.

## Code availability

The software used in the analysis is freely available and detailed in the methods section.
