# Supplementary material for: Reference SVA insertion polymorphisms are associated with Parkinson’s Disease progression and differential gene expression
Source: NPJ Parkinsons Dis. 2021 May 25;7:44. doi: 10.1038/s41531-021-00189-4 (PMC8149882; doi:10.1038/s41531-021-00189-4)
Supplement: Supplementary file 1 — Supplementary Information [file 41531_2021_189_MOESM1_ESM.pdf]

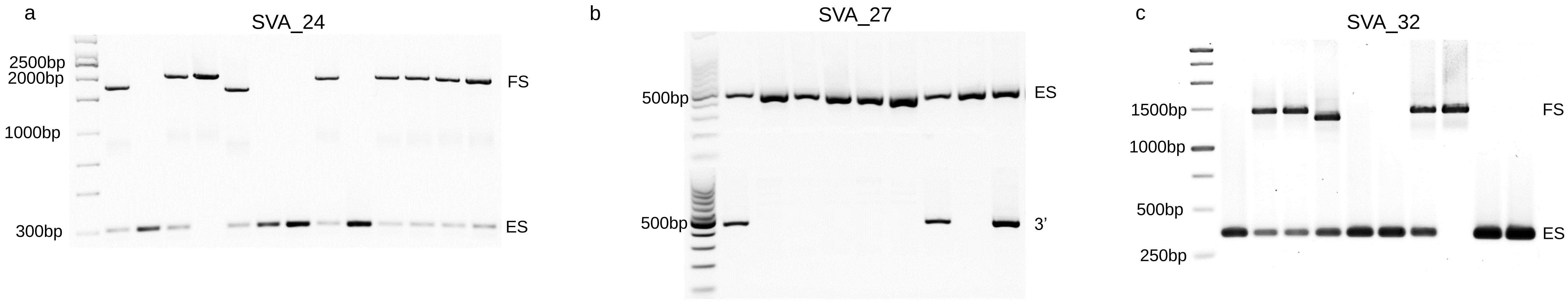

Supplementary Figure 1: Representative gel images of PCR amplification of reference SVAs the PPMI cohort. A - PCR amplification of the empty and filled site of SVA_24 upstream of the HLA-A gene. Different sized products for the present allele (filled site) of the SVA_24 demonstrates this SVA is also polymorphic for size. Expected PCR products sizes based on the reference genome: empty site – 304bp and filled site – 2062bp. B- PCR amplification of the empty site and 3’ junction of SVA_27 upstream of the HLA-DRB1 gene. Expected PCR products sizes based on the reference genome: empty site – 431bp and 3’ junction – 423bp. C - PCR amplification of the empty and filled site of SVA_32 within an intron of the SERAC1 gene. Different sized products for the present allele (filled site) of the SVA_32 demonstrates this SVA is also polymorphic for size. Expected PCR products sizes based on the reference genome: empty site – 338bp and filled site – 1153bp. FS – filled site, ES – empty site, 3’- 3’ junction. For each panel the PCR products were processed at the same time.

SVA_32 located in intron of SERAC1 gene

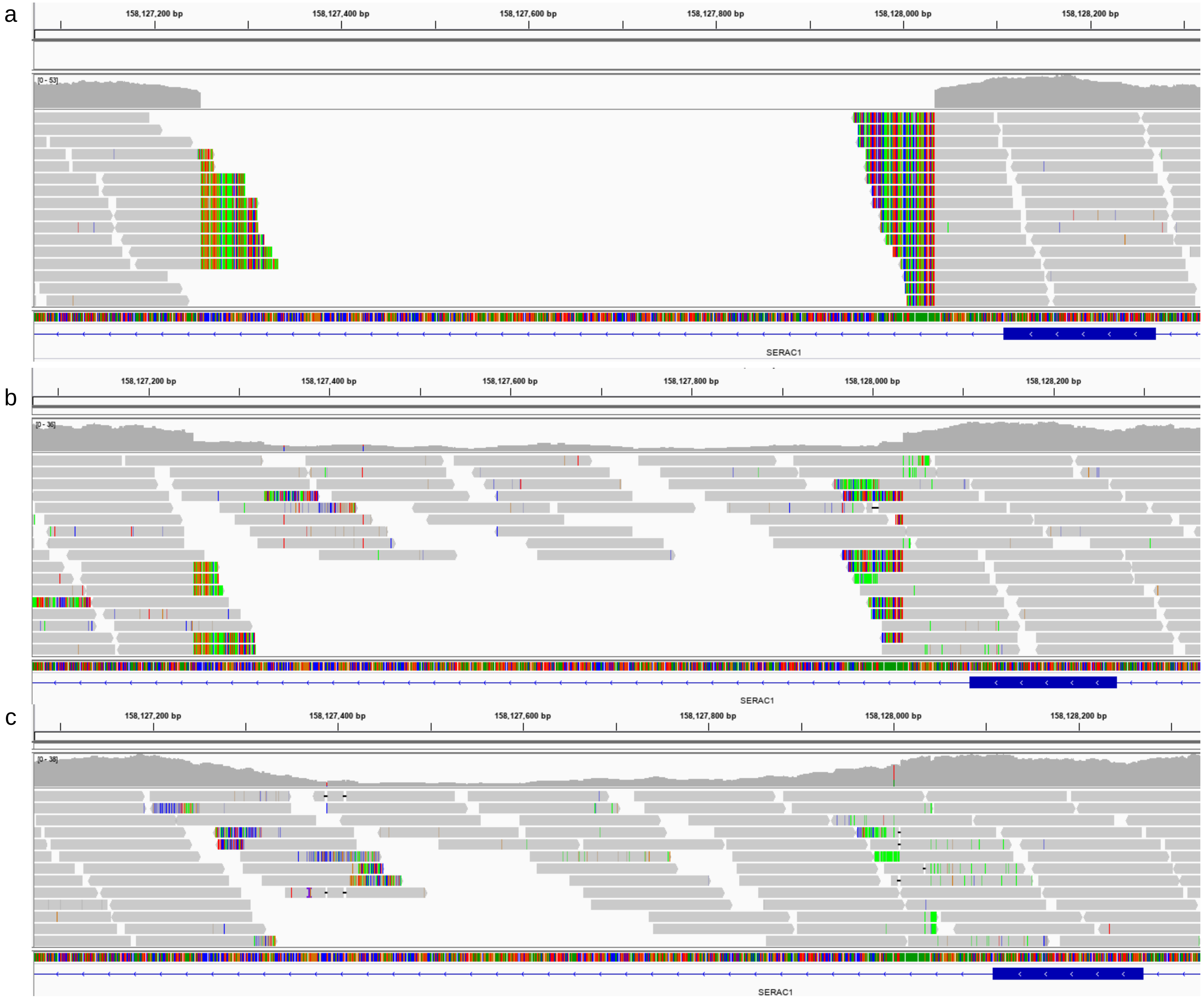

Supplementary Figure 2: Traces from whole genome sequencing bam files in IGV over SVA_32 inserted into the SERAC1 gene. A – Individual with genotype AA and the breakpoint is shown by the presence of soft clipped reads at the boundary of the SVA insertion site. B – Individual with genotype PA which is shown by the presence of soft clipped read over the breakpoint (absent allele) and those reads that map over the breakpoint (present allele). C – Individual with genotype PP shown by the presence of reads mapping over the breakpoint and lacking soft clipped reads that represent the absent allele.

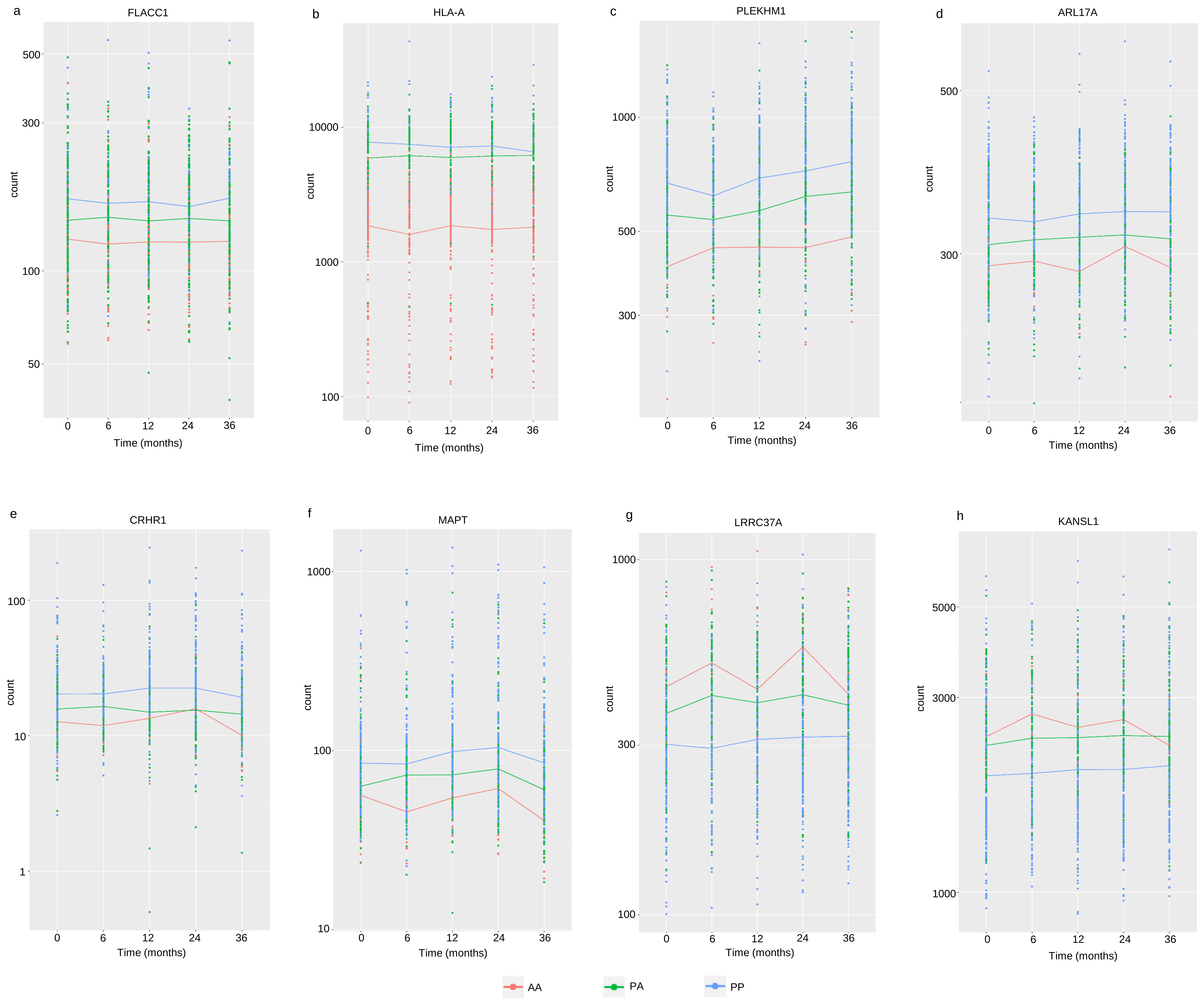

Supplementary Figure 3: Differential expression of genes associated with reference SVAs in PD subjects. A-H Expression of selected genes associated with SVA genotypes from Figure 3 and 4 for PD subjects only.

					Insertion Allele Frequency			Logistic regression (Controls vs PD)	
SVA ID	chr	start	end	dbRIP ID	Controls	PD	SWEDD	Unadjusted P value	Bonferroni corrected p value
SVA_1	1	24832213	24835019	RIP3000012	0.003	0.003	0.009	0.819	1
SVA_2	1	86215058	86217341	RIP3000043	0.963	0.962	0.940	0.868	1
SVA_3	1	113068718	113071156	RIP3000013	0.571	0.613	0.655	0.401	1
SVA_4	1	156556932	156559144	RIP3000079	0.003	0.003	0	0.858	1
SVA_5	1	203918556	203920759		0.983	0.999	1	0.029	1
SVA_6	1	212297812	212299275	RIP3000014	0.422	0.378	0.455	0.209	1
SVA_7	1	248676247	248677600		0.860	0.888	0.940	0.191	1
SVA_8	2	41746017	41748725	RIP3000047	0.275	0.318	0.193	0.090	1
SVA_9	2	48554714	48557729	RIP3000048	0.684	0.687	0.672	0.799	1
SVA_10	2	113393766	113396035	RIP3000049	0.497	0.473	0.464	0.424	1
SVA_11	2	201281925	201284718	RIP3000022	0.506	0.499	0.466	0.895	1
SVA_12	2	207609772	207612058	RIP3000050	0.188	0.157	0.155	0.239	1
SVA_13	2	216225221	216227776	RIP3000021	0.106	0.133	0.123	0.387	1
SVA_14	2	222895474	222897954	RIP3000051	0.201	0.229	0.250	0.481	1
SVA_15	3	42796882	42799319	RIP3000080	0.003	0.001	0	0.749	1
SVA_16	3	58549051	58551598		0.947	0.954	0.948	0.334	1
SVA_17	3	133784939	133786239	RIP3000081	0.629	0.698	0.672	0.053	1
SVA_18	3	194400435	194401193	RIP3000023	0.757	0.752	0.784	0.605	1
SVA_19	4	63917881	63918837		0.315	0.332	0.233	0.462	1
SVA_20	4	183734681	183737815		0.785	0.780	0.741	0.842	1
SVA_21	5	137686890	137688198	RIP3000025	0.233	0.236	0.328	0.883	1
SVA_22	5	176960578	176963180	RIP3000053	0.469	0.519	0.483	0.139	1
SVA_23	6	5037016	5038952	RIP3000055	0.240	0.232	0.164	0.488	1
SVA_24	6	29932006	29933750		0.302	0.285	0.237	0.681	1
SVA_25	6	31243854	31245339		0.155	0.122	0.149	0.202	1
SVA_26	6	31453720	31456610		0.466	0.461	0.483	0.837	1
SVA_27	6	32594189	32596833	RIP3000082	0.147	0.156	0.155	0.352	1
SVA_28	6	33525378	33526176		0.980	0.984	0.981	0.435	1
SVA_29	6	56893566	56896149	RIP3000083	0.766	0.750	0.853	0.398	1
SVA_30	6	71265416	71267495	RIP3000084	0.986	0.978	0.983	0.456	1
SVA_31	6	125387620	125390207	RIP3000056	0.688	0.695	0.629	0.580	1
SVA_32	6	158127251	158128035	RIP3000030	0.168	0.132	0.172	0.180	1
SVA_33	7	1145443	1148018	RIP3000031	0.347	0.365	0.377	0.584	1
SVA_34	7	10464690	10466507	RIP3000032	0.696	0.650	0.759	0.460	1
SVA_35	7	55219066	55221368	RIP3000033	0.489	0.519	0.482	0.386	1
SVA_36	7	56150058	56152555		0.701	0.715	0.733	0.562	1
SVA_37	7	64855447	64857680	RIP3000085	0.003	0.000	0.000	0.999	1
SVA_38	7	66392674	66395117	RIP3000060	0.600	0.600	0.588	0.781	1
SVA_39	7	101357986	101360515	RIP3000034	0.672	0.654	0.640	0.532	1
SVA_40	7	138611712	138612464	RIP3000058	0.342	0.301	0.316	0.143	1
SVA_41	7	152584622	152587221	RIP3000061	0.492	0.474	0.500	0.831	1
SVA_42	8	51533478	51535744	RIP3000063	0.997	0.992	0.970	0.289	1
SVA_43	9	33423379	33424657	RIP3000036	0.775	0.764	0.733	0.523	1
SVA_44	9	81709455	81712011	RIP3000037	0.212	0.183	0.190	0.312	1
SVA_45	9	88244082	88246049		0.106	0.101	0.052	0.861	1
SVA_46	9	107255996	107258568	RIP3000086	0.698	0.720	0.724	0.320	1
SVA_47	9	107271165	107273168	RIP3000038	0.028	0.030	0.060	0.495	1
SVA_48	9	107775255	107778315		0.226	0.199	0.267	0.316	1
SVA_49	10	6055424	6055905		0.064	0.038	0.052	0.013	1
SVA_50	10	12518475	12520975		0.506	0.485	0.474	0.642	1
SVA_51	10	31968968	31971685		0.441	0.459	0.474	0.544	1
SVA_52	10	82478234	82478710		0.265	0.263	0.284	0.664	1
SVA_53	10	92374843	92377897		0	0	0		
SVA_54	10	92673705	92676020		0.978	0.982	0.983	0.635	1
SVA_55	10	97275106	97277641		0	0.001	0	0.999	1
SVA_56	11	93946079	93948258		0.600	0.612	0.629	0.311	1
SVA_57	12	48331699	48334598		0.650	0.658	0.595	0.785	1
SVA_58	12	66133872	66136096	RIP3000089	0.566	0.550	0.647	0.825	1
SVA_59	12	70201085	70203798	RIP3000002	0.292	0.295	0.328	0.699	1
SVA_60	12	95839815	95842550	RIP3000090	0.163	0.209	0.202	0.162	1
SVA_61	12	95946567	95949177	RIP3000091	0.382	0.395	0.448	0.795	1
SVA_62	14	22638796	22640032	RIP3000093	0.789	0.774	0.784	0.671	1
SVA_63	14	35038510	35040302	RIP3000005	0.846	0.830	0.810	0.470	1
SVA_64	15	68133683	68136590		0	0	0		

SVA_65	17	18887841	18888851	RIP3000007	0.424	0.460	0.535	0.161	1
SVA_66	17	28455395	28456478	RIP3000094	0.514	0.505	0.431	0.844	1
SVA_67	17	46237519	46238226		0.778	0.793	0.776	0.685	1
SVA_68	17	66268940	66269997	RIP3000008	0.749	0.747	0.767	0.952	1
SVA_69	17	80601573	80602473		0.556	0.554	0.609	0.504	1
SVA_70	18	54342407	54344600		0.924	0.941	0.922	0.079	1
SVA_71	19	21720248	21722766		0.585	0.571	0.578	0.703	1
SVA_72	19	40107198	40109854	RIP3000095	0.844	0.822	0.767	0.344	1
SVA_73	19	40117180	40119396		0.978	0.961	0.974	0.126	1
SVA_74	19	40647368	40650100		0.829	0.875	0.842	0.030	1
SVA_75	19	41102821	41104286		0.910	0.889	0.879	0.269	1
SVA_76	19	52592770	52594354	RIP3000010	0.551	0.557	0.534	0.853	1
SVA_77	19	53185934	53188699	RIP3000011	0.011	0.009	0	0.800	1
SVA_78	20	32723075	32724969	RIP3000096	0.243	0.231	0.245	0.617	1
SVA_79	20	54249714	54250804	RIP3000045	0.980	0.979	0.990	0.978	1
SVA_80	22	23853746	23856319	RIP3000018	0.264	0.284	0.246	0.460	1
SVA_81	22	23936390	23937906		0.274	0.271	0.324	0.848	1
SVA_82	22	26772103	26773830	RIP3000017	0.374	0.375	0.440	0.909	1
SVA_83	22	40744123	40746631	RIP3000019	0.813	0.796	0.802	0.434	1

Supplementary Table 1: List of 83 reference SVAs detected as absent in the PPMI whole genome sequencing data, their chromosomal coordinates, dbRIP ID if reported previously, the insertion allele frequency in controls, PD and SWEDD samples and p values from logistic regression analysis.

Supplementary Table 2: Delly is more accurate than MELT-del for calling reference SVA genotypes based on PCR validation. SVAs were genotyped by both Delly and MELT-del in the PPMI cohort and the number of individuals in which the two callers agreed on the genotype is reported. The agreement between each caller and subsequent PCR validation is also reported. Na- not applicable as SVA_24 was not called by MELT-del

	Genotypes in Agreement (%)		
SVA ID	Delly and MELT-del	Delly and PCR	MELT-del and PCR
SVA_24	na	541/546 (99.1%)	na
SVA_27	564/605 (93.2%)	179/180 (99.4%)	167/183 (91.3%)
SVA_32	586/607 (96.5%)	179/182 (98.4%)	175/183 (95.6%)

SVA ID	Tagging SNP ID	r2 (SVA and SNP)	A1	A2	SNP summary data from Meta-analysis Nalls et al 2019 freq	b	se	p	N_cases	N_controls
SVA_1	rs60996352	1	A	C	0.0012	1.548	1.0764	0.1504	2110	1333
SVA_2	rs78105006	1	A	T	0.0309	-0.0212	0.0539	0.6947	33674	449056
SVA_3	rs1766862	0.992	T	C	0.6154	-0.0202	0.0229	0.3777	26421	442271
SVA_4	tagging SNPs not present									
SVA_5	rs9326605	1	A	G	0.9883	0.109	0.1457	0.4544	12220	10699
SVA_6	rs376245	0.992	A	G	0.3816	-0.019	0.023	0.4105	26421	442271
SVA_7	rs72769047	1	A	G	0.1166	-0.0409	0.0268	0.127	33674	449056
SVA_8	rs4331557	0.892	C	G	0.6787	0.0282	0.024	0.2407	26421	442271
SVA_9	rs6737732	0.996	T	C	0.3138	0.0231	0.0186	0.216	33674	449056
SVA_10	rs6704835	0.996	A	G	0.5183	0.004	0.017	0.8127	33674	449056
SVA_11	no tagging SNP with r2>0.8									
SVA_12	rs2551950	1	A	C	0.8168	-0.0034	0.0217	0.8742	33674	449056
SVA_13	rs207827	1	A	T	0.8737	-0.0057	0.0337	0.8648	26421	442271
SVA_14	rs795887	0.994	T	C	0.7676	0.005	0.0266	0.8524	26421	442271
SVA_15	rs7648688	1	T	C	0.9971	0.748	0.4224	0.07657	2985	1780
SVA_16	rs28449395	1	A	G	0.9577	0.0217	0.047	0.6444	33674	449056
SVA_17	rs1830083	0.996	A	T	0.6038	-0.0194	0.0193	0.313	33674	449056
SVA_18	rs1466733	0.948	A	G	0.7412	0.002	0.0257	0.9384	26421	442271
SVA_19	rs12639620	1	A	T	0.329	-0.0055	0.0235	0.814	26421	442271
SVA_20	rs35791833	1	A	G	0.7816	-0.0151	0.0269	0.574	26421	442271
SVA_21	rs2905591	0.994	T	C	0.2344	1.00E-04	0.0256	0.9984	27823	443190
SVA_22	rs353503	1	T	G	0.4932	0.0302	0.022	0.1708	26948	442743
SVA_23	rs726743	0.995	A	G	0.7812	-0.0401	0.0273	0.1424	26421	442271
SVA_24	rs9259888	0.988	T	C	0.3509	0.0314	0.0285	0.2709	13887	11669
SVA_25	rs75899922	1	A	G	0.8657	0.0251	0.0276	0.3648	32505	448088
SVA_26	rs2516453	1	A	T	0.4436	-0.0036	0.0192	0.8507	32505	448088
SVA_27	rs9270656	1	A	C	0.8384	6.00E-04	0.0264	0.9828	32505	448088
SVA_28	rs58020942	1	T	G	0.0115	0.1816	0.0949	0.05562	13887	11669
SVA_29	rs9475744	0.989	A	G	0.7378	-0.0282	0.0227	0.2151	33674	449056
SVA_30	rs113160712	0.955	T	C	0.0185	0.0585	0.0771	0.4478	33162	448774
SVA_31	rs6933645	0.979	A	G	0.3073	-0.0036	0.0242	0.8803	26421	442271
SVA_32	rs1951543	0.985	A	G	0.1329	-0.024	0.0291	0.4109	33674	449056
SVA_33	rs7805362	0.952	A	G	0.3771	-0.0171	0.0201	0.3949	33674	449056
SVA_34	rs1524237	0.988	C	G	0.3266	-0.0168	0.0238	0.4804	26421	442271
SVA_35	rs6952602	0.899	C	G	0.5174	-0.0266	0.0223	0.2345	26421	442271
SVA_36	rs816228	0.982	A	G	0.3241	-0.0404	0.0239	0.0911	26421	442271
SVA_37	rs58376065	1	C	G	0.006	-0.3857	0.2793	0.1672	3973	2715
SVA_38	rs778706	0.971	A	G	0.5791	0.0183	0.0176	0.2988	33674	449056
SVA_39	no tagging SNP with r2>0.8									
SVA_40	rs1673222	0.991	A	G	0.324	-0.026	0.0237	0.2729	26421	442271
SVA_41	rs6967158	1	A	C	0.4846	-0.0047	0.0263	0.8592	7803	5852
SVA_42	rs114111969	1	A	T	0.009	-0.0931	0.1965	0.6355	5865	4689
SVA_43	rs60636152	1	C	G	0.7893	0.0229	0.0245	0.3506	33674	449056
SVA_44	rs2488274	0.890	C	G	0.7885	0.0087	0.0278	0.7546	26421	442271
SVA_45	rs7037921	1	A	G	0.094	-0.0254	0.0374	0.4963	27823	443190
SVA_46	rs2417776	1	T	G	0.276	-3.00E-04	0.0248	0.9894	26421	442271
SVA_47	rs10733562	0.948	C	G	0.9626	-0.0885	0.0593	0.1357	26421	44227
SVA_48	rs1357574	0.995	C	G	0.2316	-0.0441	0.0263	0.09348	26421	442271
SVA_49	rs1323658	0.924	A	C	0.9435	0.0384	0.0481	0.4245	27823	443190
SVA_50	rs2815646	0.996	T	C	0.5223	0.0378	0.0222	0.08945	26421	442271
SVA_51	rs1615256	1	T	C	0.4541	-0.001	0.018	0.9569	33674	449056
SVA_52	rs4933837	0.981	T	C	0.2588	0.0095	0.0215	0.6571	33674	449056
SVA_53	monomorphic in PPMI cohort									
SVA_54	rs147194420	1	T	C	0.9792	-0.0379	0.0798	0.6349	27823	443190
SVA_55	no tagging SNP with r2>0.8									
SVA_56	rs7940973	1	C	G	0.5975	-0.031	0.0178	0.08197	33674	449056

SVA_57	rs2732441	1	C	G	0.6615	-0.0295	0.0185	0.1105	32505	448088
SVA_58	rs1177578	0.992	C	G	0.4273	0.0194	0.0174	0.2635	33674	449056
SVA_59	rs7954979	0.996	A	G	0.685	-0.0423	0.0213	0.04692	33674	449056
SVA_60	rs7299632	1	A	C	0.8032	-0.0234	0.0275	0.3945	27823	443190
SVA_61	rs7486703	1	A	G	0.4235	-0.001	0.0214	0.9645	27460	443025
SVA_62	rs1681589	0.891	T	C	0.7612	-0.0048	0.026	0.8522	26421	442271
SVA_63	rs78207779	1	T	C	0.8314	-0.0189	0.0296	0.5222	26421	442271
SVA_64	monomorphic in PPMI cohort									
SVA_65	rs4924938	1.000	T	C	0.44	0.0143	0.017	0.4	33674	449056
SVA_66	rs4555192	0.996	A	C	0.4877	0.0142	0.018	0.4297	32505	448088
SVA_67	rs55653937	0.994	T	G	0.2157	-0.2488	0.0275	1.64E-19	32505	448088
SVA_68	rs9910520	1	T	C	0.7465	-0.014	0.0195	0.4737	33674	449056
SVA_69	rs8071934	0.969	T	C	0.4152	0.0512	0.0224	0.02234	26421	442271
SVA_70	rs1657882	1	A	G	0.0602	0.0054	0.0351	0.8787	33674	449056
SVA_71	rs61037485	0.992	A	T	0.5668	0.0285	0.0173	0.09899	33674	449056
SVA_72	rs186816	0.987	T	C	0.186	0.0011	0.0246	0.9638	33674	449056
SVA_73	rs2767591	1	A	G	0.9688	-0.0528	0.0523	0.3122	33674	449056
SVA_74	rs34652347	0.992	T	G	0.1354	0.0089	0.0276	0.7475	33674	449056
SVA_75	rs145706893	1	T	G	0.8838	0.0017	0.0346	0.9601	27460	443025
SVA_76	rs7259905	1	A	G	0.5618	0.0034	0.0223	0.8779	27460	443025
SVA_77	rs7255114	1	A	C	0.012	0.1311	0.1219	0.2818	13524	11504
SVA_78	rs6058828	1	A	G	0.2311	0.0245	0.0264	0.3529	26421	442271
SVA_79	rs113448067	1	A	T	0.9786	0.0144	0.0834	0.8627	26421	442271
SVA_80	no tagging SNP with r2>0.8									
SVA_81	no tagging SNP with r2>0.8									
SVA_82	rs5752409	1	A	G	0.3739	-0.0187	0.0231	0.4171	26421	442271
SVA_83	rs9611403	1	T	C	0.2043	-0.0162	0.0239	0.4969	33674	449056

Supplementary Table 3: Summary data from published PD meta-analysis for all SNPs identified as tags for the polymorphic reference SVAs in this study.

Supplementary Table 4: List of 111 clinical variables from the PPMI cohort analysed in the linear mixed effects model.

primdiag	quip_walk	ips_striatum
hy	quip	ips_caudate
hy_on	quip_any	ips_putamen
NHY	rem	ips_cdr
NHY_ON	rem_cat	urate
rigidity	rem_q6	LEDD
rigidity_on	scopa_gi	hemo
td_pigd	scopa_ur	abeta
td_pigd_on	scopa_cv	asyn
td_pigd_old	scopa_therm	tau
td_pigd_old_on	scopa_pm	ptau
tremor	scopa_sex	tau_ab
tremor_on	scopa	ptau_ab
updrs3_score	VLTANIM	ptau_tau
updrs3_score_on	VLTVEG	ab_asyn
MSEADLG	VLTFRUIT	tau_asyn
updrs1_score	sft	ptau_asyn
NP1COG	stai_state	changedx
NP1HALL	stai_trait	hemohi
NP1DPRS	stai	
NP1ANXS	SDMTOTAL	
NP1APAT	moca	
NP1DDS	CAUDATE_R	
NP1FATG	CAUDATE_L	
updrs2_score	PUTAMEN_R	
updrs_totscore	PUTAMEN_L	
updrs_totscore_on	r_striatum	
upsit	l_striatum	
bjlot	mean_caudate	
ess	mean_putamen	
ess_cat	mean_striatum	
gds	low_caudate	
gds_cat	hi_caudate	
hvlt_immediaterecall	low_putamen	
HVLTRDLY	hi_putamen	
HVLTREC	low_striatum	
HVLTFPRL	hi_striatum	
hvlt_discrimination	ai_caudate	
hvlt_retention	ai_putamen	
lns	ai_striatum	
quip_gamble	l_cdr	
quip_sex	r_cdr	
quip_buy	con_striatum	
quip_eat	con_caudate	
quip_hobby	con_putamen	
quip_pund	con_cdr	

Supplementary 5: The number of PD subjects analysed at each time point in the longitudinal analysis.

SVA_7				
Months	0	12	24	36
AA	2	2	2	2
PA	79	76	74	72
PP	289	266	254	248

SVA_11				
Months	0	12	24	36
AA	67	61	58	58
PA	229	213	205	202
PP	66	62	59	56

SVA_24				
Months	0	12	24	36
AA	194	179	168	165
PA	130	123	118	114
PP	38	36	36	36

SVA_36				
Months	0	12	24	36
AA	29	26	25	26
PA	150	138	134	129
PP	186	176	166	162

SVA_47				
Months	0	12	24	36
AA	348	323	308	301
PA	16	16	15	15
PP	3	2	3	3

SVA_67				
Months	0	12	24	36
AA	13	13	11	12
PA	126	116	112	109
PP	229	213	204	199

SVA_79				
Months	0	12	24	36
PA	13	13	13	12
PP	295	276	266	256
